# Correlation of Viral Loads with HCV Genotypes: Higher Levels of Virus Were Revealed among Blood Donors Infected with 6a Strains

**DOI:** 10.1371/journal.pone.0052467

**Published:** 2012-12-17

**Authors:** Xia Rong, Ling Lu, Junzhi Wang, Huaping Xiong, Jieting Huang, Jinyan Chen, Ke Huang, Ru Xu, Min Wang, Xuemei Zhang, Tai Guo, Yueyue Liu, Guoquan Gao, Yongshui Fu, Kenrad E. Nelson

**Affiliations:** 1 Department of Biochemistry, Medical College of Sun Yat-sen University, Guangzhou, Guangdong, China; 2 Guangzhou Blood Center, Guangzhou, Guangdong, China; 3 Center for Viral Oncology, University of Kansas Medical Center, Kansas City, Kansas, United States of America; 4 National Institutes for Food and Drug Control, Beijing, China; 5 Department of Epidemiology, Bloomberg School of Public Health, Johns Hopkins University, Baltimore, Maryland, United States of America; University of Kansas Medical Center, United States of America

## Abstract

**Background:**

Both HCV genotypes and viral loads are predictors of therapeutic outcomes among patients treated with α-interferon plus ribavirin; however, such correlation has only been studied for genotypes 1, 2, and 3 but not for genotype 6.

**Methodology/Findings:**

299 voluntary blood donors were recruited who were HCV viremic. Their mean age was 31.8; the male/female ratio was 3.82 (225/59). The viral loads of HCV were measured using the COBAS AmpliPrep/COBAS TaqMan test (CAP/CTM) while HCV genotypes were determined by direct sequencing the partial NS5B region. HCV genotypes 1, 2, 3, and 6 were determined in 48.9%, 8.7%, 12.3%, and 30.1% of the donors, respectively, and the levels of mean viral loads in genotype 1 and 6 significantly higher than that of 2 and 3 (*P*<0.001). As a whole, the viral loads in male donors were higher than in female (P = 0.006). Moreover, the donors' gender and HCV genotypes are independently correlated with the measured viral loads.

**Conclusion:**

HCV genotype 1 and 6 had significantly higher viral loads than genotype 2 and 3.

## Introduction

Hepatitis C virus (HCV) is a blood-borne pathogen that has imposed a serious global health problem. Currently, an estimated 130–170 million people, i.e. about 3% of the world's population, are chronically infected with the virus and over 350,000 patients die from the HCV-related liver diseases annually which include liver cirrhosis and hepatocellular carcinoma (HCC) [1], [2]. According to a report from the World Health Organization (WHO), countries that have high rates of HCV infection included Egypt (22%), Pakistan (4.8%), and China (3.2%) [2], [3]. Other studies have reported high HCV prevalence in Thailand (5.6%) and Vietnam (6.1%) [4], [5].

Analysis of viral sequences has resulted in the classification of HCV into six major genotypes and over 80 subtypes [6], and different genotypes have shown varied patterns of geographic distribution. Generally, genotypes 1a, 1b, 2a, 2b, and 3a are worldwide epidemic [7], [8], [9]. In contrast, genotype 4 is often found in North Africa and the Middle East [10], 5a in South Africa [11], and genotype 6 in Southeast Asia [12]. HCV genotypes are an important factor for patients' management because their variations are associated with different responses to the therapy with pegylated interferon plus ribarivin – the current standard regimen for treating chronic hepatitis C [13], [14], [15]. Although less understood, viral load may be another factor that affects the treatment duration, dosage, and responses [15], [16], [17]. It has been argued that viral load may be an outcome of the genotype-specific variation but does not affect treatment [18]. Studies have shown that patients infected with genotype 1 had higher viral loads than those infected with genotype 2 or 3 [19], [20], [21]. However, correlations between viral loads and other HCV genotypes have not been described.

We have recently reported that subtype 6a accounted for 34.8% of the HCV infected blood donors in China [12]. Given the high prevalence and rapid dissemination of these viral strains, there is still an insufficiency of studies in addressing their clinical features. It has been described that patients infected with HCV genotype 1 and 6 in Hong Kong showed comparable levels of viral RNA in serum [22], [23]. Other studies that focused on Asian American patients have also implied that patients infected with genotype 1 or 6 had similar viral loads, while among patients infected with genotype 6 and genotype 2/3 the levels of HCV RNA were different [24], [25]. Regardless, all these studies were limited by small sample sizes and there is a need for more studies involving a larger number of cohort.

The aim of the present study was to determine the correlation between HCV genotypes and viral loads in plasma samples from blood donors who were HCV viremic, particularly among those infected with genotype 6. For this aim, 299 voluntary blood donors were recruited who were HCV viremic. For these donors, the viral loads in plasma were measured using the COBAS AmpliPrep/COBAS TaqMan assay (CAP/CTM) while the genotypes were determined by sequencing. The results should shed lights on the clinical and virological aspects of HCV genotype 6.

## Materials and Methods

### Subjects and samples

All plasma samples were collected from voluntary blood donors recruited at the Guangzhou Blood Center from November 2009 to August 2011. Before blood donation, individuals were informed to complete a Blood Donation Healthy Consulted form. For donors privacy we can't disclose the form. HCV, HBV, HIV and TP assays were performed for blood screening and the anti-HCV-positive samples were informed to participate in this study. The physicians ensured that individuals were personally interviewed to assure their complete understanding of the informed consent and the participants provided their verbal informed consent by telephone. The study protocol conformed to the ethical guidelines of the 1975 Declaration of Helsinki and was approval by Medical Ethics Committee of Guangzhou Blood center. After routine but mandatory screening, 707 donors were found to be anti-HCV positive. Of them, 527 had sufficient volumes for Nucleic Acid Testing of HCV (NAT), which gave positive results for 302 donors. These latter 302 donors were then subjected to HCV RNA quantification using the COBAS AmpliPrep/COBAS TaqMan test (CAP/CTM), for which the positive range was set from 43 to 6.9×10^7^ international unit (IU)/ml. Since three samples had HCV RNA levels lower than 43 IU/ml, they were discarded. Thus, 299 samples remained and were regarded as HCV RNA positive, for which HCV genotypes were further determined by sequencing. Methods for the Anti-HCV assay and NAT followed those previously described [26]. This study has been approved by the Institutional Review Board at the Guangzhou Blood Center and guidelines set by this board were strictly followed.

### HCV genotyping

HCV genotypes were determined as previously described [27]. In brief, partial NS5B or E1 region sequences were amplified using the Primer STAR kit (Takara, Dalian, China). Among the 299 donors, 298 were amplified successfully and 1 was failed by NS5B primer. And then the only one was amplified by E1 primer. Amplicons were sequenced in both directions on an ABI Prism 3100 genetic analyzer (PE Applied Biosystems, FosterCity, CA, USA). Sequences were aligned using the CLUSTAL_X program (www.geneious.com). Phylogenies were estimated using the maximum-likelihood method under the HKY+I+Γ_6_ substitution model in the MEGA5 (http://www.megasoftware.net/mega.php). Bootstrap resampling was performed in 1000 replicates. Reference sequences used for analyses were retrieved from Genbank (Table S1).

### Nucleotide sequence accession numbers

The nucleotide sequences reported in this study were deposited into Genbank with the following accession numbers: GenBank JX521873-JX522171.

### Determination of HCV load in plasma

Viral loads of HCV in plasma were measured by the CAP/CTM test (Roche Molecular Systems, Inc., Branchburg, NJ) using the published methods [28]. In brief, 1ml of plasma was applied to the automated Cobas Ampliprep Instrument for RNA extraction. This was followed by an automated real-time PCR amplification and detection using the Cobas TaqMan 48 analyzer. The generated data were analyzed using the Amplilink software. HCV load in plasma was expressed as log_10_ international units per milliliter (log_10_ IU/ml).

### Statistical analyses

Firstly, chi-squared test was used to analyze the correlations between genotype, age, ethnicity, and gender. Secondly, since there are four genotype groups, analysis of variance was applied to compare the viral loads among these groups. Meanwhile, T-test was employed to compare the viral loads between the male, female, Han, and non-Han groups. Lastly, to further detect the true factors that affect the viral loads in the genotype 6 group, multivariate regression analysis was performed. In all the analyses described above, any test with p value less or equal to 0.05 was indicated to be statistically significant. All these statistical analyses were performed using SPSS for Windows, version 16.0 (SPSS, Chicago, IL, USA).

## Results

### Detected HCV genotypes

HCV genotypes were determined among the 299 donors who were HCV viremic. Among them 173 (57.9%) had origins in Guangdong province, 121 (40.5%) in areas other than Guangdong, while the birthplaces for five (1.7%) were unknown. Table 1 and Figure S1 showed the patterns of HCV genotype distribution among these 299 donors: 1b and 6a were predominant (48.2% and 30.1%, respectively), followed by 2a (8.7%), 3a (8.0%), 3b (4.3%), and 1a (0.7%). Among those having origins in Guangdong province, the frequencies of 1b (43.4%) and 6a (38.2%) were comparable, while among those having origins in areas other than Guangdong, the proportions of 1b (55.4%) and 6a (19.0%) were remarkably different. These patterns resembled that we have recently described [12]. Once again it was verified that 6a has become a major HCV strain in China, particularly in Guangdong province.

**Table 1:** HCV genotype distribution (%).

Subtype		1a	1b	2a	3a	3b	6a	Total
		2(0.7)	144(48.2)	26 (8.7)	24 (8.0)	13(4.3)	90(30.1)	299(100.0)
	Guangdong	1(0.6)	75 (43.4)	6 (3.5)	19(11.0)	6 (3.5)	66(38.2)	173(100.0)
Birthplace	Other areas	1(0.8)	67 (55.4)	18(14.9)	5 (4.1)	7 (5.8)	23(19.0)	121(100.0)
	Missing data		2	2			1	5

### Donors' demographic characteristics

Based on the detected HCV genotypes, the blood donors were divided into four groups with each group being represented by one genotype: genotype 1, 2, 3, and 6, respectively. In addition, according to the donors' gender and their ethnic origins, each genotype group was further divided into the male, female, Han, and non-Han groups (Table 2). Since 15 donors lacked these pieces of information, only 284 donors were here analyzed. Based on the policy of voluntary blood donation, only donors aged from 18–55 years were recruited. Thus, the donors' overall mean age was 31.8. Among the genotype 1, 2, 3, and 6 groups, the mean ages were 30.2, 29.7, 33.4, and 34.2, respectively, and no statistical significance was shown (χ^2^ = 4.936, *P* = 0.177). Of the 284 donors analyzed, 274 (96.5%) were of Han origin while 10 (3.5%) of minority ethnicities. Although among the four genotype groups similar ethnic compositions were observed (χ^2^ = 1.864, P = 0.601), their gender ratios were significantly different (χ^2^ = 9.352, *P* = 0.025). Among the genotype 1, 2, and 3 groups, the male percentages were similar (73.9%, 73.9% and 75.7%, respectively), but they were significant lower than the percentage in the genotype 6 group (90.0%). Taken together, among the four genotype groups and excluding gender, the donors' age and ethnicity were statistically matched.

**Table 2:** General information of the studied donors among genotype groups.

Genotype groups		1 n = 134	2 n = 23	3 n = 37	6 n = 90	Total n = 284	*P*-value
Age	Mean ± SD	30.2±10.1	29.7±9.9	33.4±7.0	34.2±7.5	31.8±9.1	0.177
Gender (%)	Male	99 (73.9)	17 (73.9)	28 (75.7)	81 (90.0)	225 (79.2)	0.025
	Female	35 (26.1)	6 (26.1)	9 (24.3)	9 (10.0)	59 (20.8)	
Ethnicity (%)	Han	131 (97.8)	22 (95.7)	36 (97.3)	85 (94.4)	274 (96.5)	0.601
	Others	3 (2.2)	1 (4.3)	1 (2.7)	5 (5.6)	10 (3.5)	

### Correlation between viral loads, HCV genotypes, and the donors' gender

**Figure 1:**
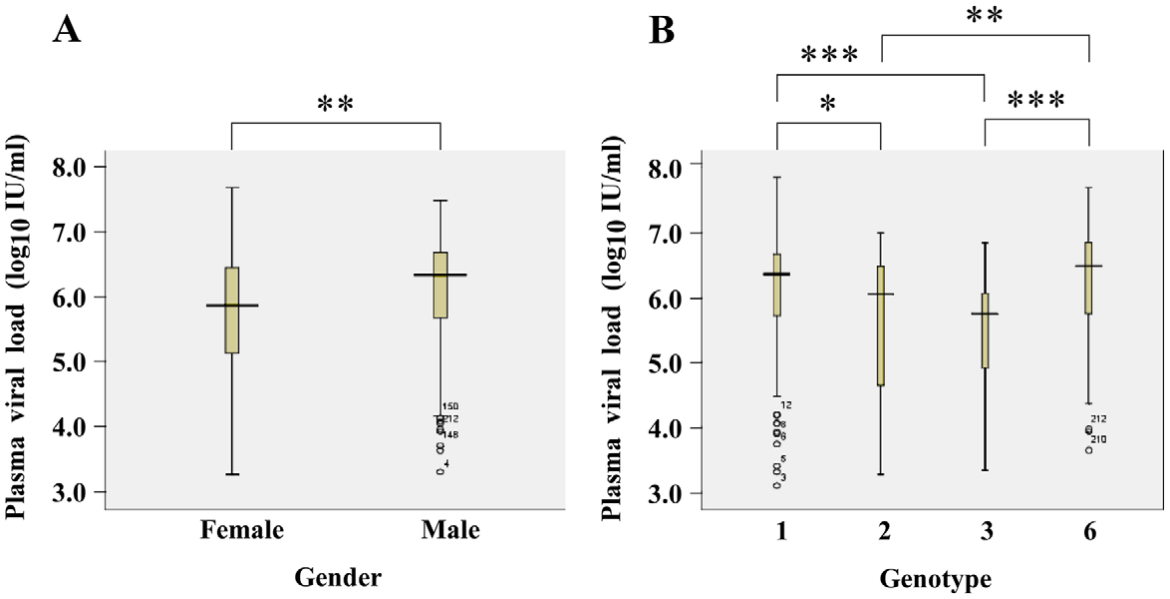
Comparing the viral loads of HCV in plasma by gender and detected HCV genotypes. Box plots of plasma HCV loads were shown in relation with gender (A) and genotype (B). The line through the box represents the mean viral load of the group. The top and bottom of the box are 25^th^ and 75^th^ percentiles, while vertical lines from the ends of the box encompass the extreme data. * *P*<0.05, ** *P*<0.01, *** *P*<0.001.

Univariate analysis was performed to analyze the correlation between the viral loads and the detected HCV genotypes. The detail viral loads data can be found in Table S2. Since there was a higher proportion of male donors in the genotype 6 group while the male gender has been reported to be a risk factor for sustaining higher levels of the virus [29], a correlation between the viral loads and the donors' gender was also analyzed. As shown in Table 3 and Figure 1, statistical analyses revealed that the viral loads were significantly different between the male and female donors and among the four genotype groups. The mean viral load among male donors was 6.06 log_10_ IU/ml comparing to 5.69 log_10_ IU/ml among female donors (t = 2.785, *P* = 0.006). Among the four genotype groups, significant differences in viral loads were also found (F = 6.675, *P*<0.001). Donors infected with genotype 1 and genotype 6 showed higher mean viral loads, which were 6.07 and 6.15 log_10_ IU/ml, respectively. However, among donors infected with genotype 2 and genotype 3 the viral loads were lower, which were 5.66 and 5.49 log_10_ IU/ml, respectively. These results indicated that male donors and donors infected with genotype 1 and 6 were more likely to have higher loads of HCV.

**Table 3:** Univariate analysis of the viral load of HCV in plasma by gender and genotypes.

Factor		Viral load (log 10) Mean ± SD	t/F-value	*P*-value
Gender	Male (n = 225)	6.06±0.87	t = 2.785	0.006
	Female (n = 59)	5.69±1.01		
Genotypes	1 (n = 146)	6.07±0.91	F = 6.675	0.0002*
	2 (n = 26)	5.66±1.06		
	3 (n = 37)	5.49±0.84		
	6 (n = 90)	6.20±0.93		

* *P*-value was calculated by one-way ANOVA, the multiple comparisons showed that genotype 1 and genotype 6 were higher than genotype 2 and genotype 3, *P*<0.05.

Higher viral loads have been reported among patients infected with genotype 1 than with genotype 2/3 [19], [20], [21]. In this study, we observed higher viral loads not only in the genotype 1 group but also in the genotype 6 group, when comparing with those in the genotype 2 and 3 groups. However, since a higher percentage of male donors was found in the genotype 6 group than in the genotype 1, 2, and 3 groups while the male gender showed higher viral loads than female gender, a question was asked if the higher viral loads we observed among the donors infected with genotype 6 was contributed mainly by HCV genotype or by donors' gender.

To answer this question, a multivariate regression analysis was performed under the generalized linear model. As shown in Table 4, both the donors' age and ethnicity were not correlated with the viral loads (*P* = 0.973 and 0.212, respectively), while the donors' gender and HCV genotype were (*P* = 0.031 and 0.011, respectively). We also compared the viral loads among male donors (Table 5). In agreement with the results previously described [19], [20], [21], the viral loads among male donors infected with genotype 1 or 6 were significant higher than those with genotype 2 or 3, with the mean values of 6.18, 6.18, 5.63, and 5.59 log_10_ IU/ml, respectively (F = 5.501, *P* = 0.001). In conclusion, both multivariate regression analysis and stratified analysis confirmed that independently the viral loads were correlated with the detected HCV genotypes and the donors' gender.

**Table 4:** Multivariate regression analysis of the viral load of HCV in plasma.

Source	Wald Chi-Square	df	Sig.
(Intercept)	979.561	1	<0.001
Age	19.231	33	0.973
Ethnicity	1.556	1	0.212
Gender	4.633	1	0.031
Genotype	11.058	3	0.011

Dependent Variable: viral load (log_10_ IU/ml) Model: (Intercept) age, ethnicity, gender, HCV genotype.

**Table 5:** Association of the viral load of HCV in plasma and genotype among male donors.

Genotype	Viral load (log 10) Mean ± SD	F-value	*P*-value
1 (n = 99)	6.18±0.74*	5.501	0.001*
2 (n = 17)	5.63±1.05		
3 (n = 28)	5.59±0.75		
6 (n = 81)	6.18±0.95*		

* *P*-value was calculated by one-way ANOVA, the multiple comparisons showed that genotype 1 and genotype 6 were higher than genotype 2 and genotype 3, *P*<0.05.

## Discussion

In this study, the viral loads of HCV were analyzed among a cohort of voluntary blood donors who were HCV viremic. It was revealed that both the detected HCV genotypes and the donors' gender are two independent factors in association with the measured viral loads. Although similar analyses have been reported for patients infected with genotype 6 comparing with those infected with genotypes 1, 2, and 3, no statistical differences were shown [24], [25]. However, in this study, the genotype 6 viruses were found to significantly associate with higher viral loads. Such a feature is similar to that revealed for genotype 1 but different from genotypes 2 and 3.

In this study, the voluntary blood donors were otherwise asymptomatic and healthy except for HCV being positive. Different from the subjects in previous studies who were patients with chronic HCV infection, none of blood donors in this study had received any anti-HCV treatment and hence, their viral loads represent those yielded during the natural HCV infection. The observed baseline values may predict the treatment outcomes and the difficulties in treating those with high HCV loads. For the viral load measurement we employed the CAP/CTM assays, while for the determination of HCV genotypes we directly sequenced the partial NS5B and E1 region. The former represents one of the most advanced real-time PCR approaches and is considered to be sensitive, specific, accurate, reproducible, and reliable, encompassing a broad range of dynamics for quantitating HCV RNA [30]. Although it has been argued for this assay to possibly underestimate the viral loads for HCV genotype 4 [30], such a genotype was rarely seen in China and completely absent in this study. Thus, the given results are thought to virtually measure the actual HCV levels. Direct sequencing partial NS5B and/or E1 region is currently the gold standard for HCV genotyping and is thus recommended for clinical use, especially for its accuracy in discriminating both genotypes 1 and 6 [9]. Another widely-used commercial kit is the INNO-LiPA (Innogenetics, Ghent, Belgium) system, which can also sensitively determine HCV genotypes but is based on the sequence differences in 5′UTR. Although more convenient and less time-consuming, this assay may incorrectly classify genotype 6 as genotype 1, since both genotypes may have identical 5′UTR sequences [9], [31].

Among the blood donors, the detected 6a strains accounted for 30.1% of the total HCV isolates. This is consistent with one of our recent reports that 6a has become local epidemic in Guangdong province and recently disseminated to other regions of China [26], [27], [32]. Other reports have also revealed that 6a is prevalent in Vietnam, Thailand, Laos, as well as in Hong Kong, Taiwan, and certain areas of China [12], [22], [33], [34]. Additional studies further revealed that genotype 6 infections were exclusively observed among Asian immigrants in Europe and North America [24], [35]. Considering the relatively high viral loads found among donors infected with 6a, we speculate that the 6a strains may replicate and propagate more efficiently among Chinese, which may help to explain why 6a has become epidemic in Guangdong and rapidly disseminated across China.

The detected HCV genotypes and viral loads are both important predictors for therapeutic outcomes and their association has been extensively analyzed. It has been reported that patients infected with genotype 1 were more likely to have higher viral loads than those infected with genotype 2 and 3 [16], [19], [20], [21]. In agreement with these reports, we also found that donors infected with genotype 1 had higher viral loads than those infected with genotype 2 and 3. At least partially, this phenomenon has been linked to lower rates of sustained virological response (SVR) among patients infected with genotype 1 who had been treated with interferon plus ribavirin [36]. In this study, we further revealed that donors infected with 6a strains tended to have similar levels of viral load to those infected with genotype 1. Although there are now only limited data available about the treatment responses among patients infected with genotype 6 [22], [23], [25], the European Association for the Study of Liver (EASL) has recommended that these patients should be treated using the strategy similar to those used in treating genotype 1 infections [17]. The EASL has also recommended that the treatment duration can be shortened if the viral load is lower than 8×10^5^ IU/ml (equals to 5.9 log_10_ IU/ml) [17]. Our results are in agreement with the EASL instructions on this, because the mean viral loads among the donors infected with genotype 1 and 6 were both higher than 6.0 log_10_ IU/ml while the viral loads among those infected with genotype 2 and 3 were lower than 5.7 log_10_ IU/ml. Nevertheless, several other studies have also reported that patients infected with genotype 6 appeared to show similar treatment responses to those infected with genotype 2/3, of which the SVR rates were both higher than that seen among patients infected with genotype 1 [22], [23], [25]. For verification, further studies are needed, which should include more patients to be matched not only with the age, gender, ethnic and geographic origins but also with HCV subtypes and basal viral loads.

Blood transfusion used to be the major risk in acquiring HCV infection prior to the institution of a mandatory anti-HCV screening [37]. Since 1992 the screening has been implemented in the United States and thus the risk has declined from 1/200 per unit of blood to 1/10,000∼1/10,000,000 [38]. Such a risk did not decline in China until the central government enacted the anti-HCV screening in 1993 and outlawed paid blood donations in 1998 [26]. With the risk via transfusion greatly decreased, the risk via injection drug use (IDU) is increasing, which has now become the major risk for contracting HCV infection in China [39]. It has been argued that sexual transmission may also be a major risk for HCV infection especially among male IDUs who have sex with men or with prostitutes [40], [41]. In addition, high viral loads has been indicated to increase the risk of HCV vertical and needlestick transmissions [42], [43]. Concurrent with a recent transition in the risk from transfusion to IDU, the prevalence of 6a is increasing while 1b is decreasing. As we know, 1b has been regarded to be more associated with HCV transmission via blood transfusion while 6a typically linked to IDU and sexual transmission [12]. In this study, all blood donors were asked to answer a standardized questionnaire before blood donations which listed all the known risk factors. Donors would be excluded when having a history of transfusion of blood or blood products, IDU, receiving a tattoo, ear or body piercing, surgery, or other invasive medical procedures. Follow-up studies were also performed on those who were HCV viremic. However, only a small proportion of the donors confessed having these risks (data not shown). It is concerning that subtype 6a might have spread to the general population via the IDU network or through illegal sexual workers. In this regard, a significantly higher proportion of male, found among donors infected with 6a than with other HCV genotypes, is implicative.

We found that the percentage of male donors who were HCV viremic is about 3.8 times as many as that of the female donors (79.2% versus 20.8%), while in initial screening a total of 707 voluntary blood donors were detected to be positive for anti-HCV among whom the male/female ratio is about 2.5 (503/204). It has been reported that women are more likely to clear the virus spontaneously after acute infection [44], [45]. This can be interpreted that men are more likely to develop chronic hepatitis than women and continue to be HCV viremic. The interpretation helps to explain why male donors tended to have higher levels of HCV RNA than female donors (6.06 versus 5.69 log 10 IU/ml), which is consistent with the results from a very recent large-scale study based on a multi-ethnic group of IDUs [29]. We firmly believe that the outcomes of HCV infection among women are much better than among men. In support of this belief, there exist additional lines of evidence: 1) HCV is more likely to infect men. In the USA, the prevalence of anti-HCV among men was twice as that among women [4]. In one of our recent studies, a significantly higher anti-HCV rate has also been revealed among male donors than among female [26]. 2) The male gender has been considered to be one of the key factors in promoting the progression of hepatic fibrosis as a result of chronic HCV infection [46]. 3) Female hormones have been identified to function as inhibitors against HCV. It has been reported that the estrogen receptor alpha (ESR1) can promote HCV replication by interaction with the NS5B protein, an RNA-dependent RNA polymerase encoded by HCV genome [47], [48], while this interaction can be abolished by 17-estradiol or tamoxifen [48], [49]. Comparing with premenopausal female patients, postmenopausal female have faster progression of hepatic fibrosis, but the latter can be delayed by hormone replacement therapy with estrogen and progesterone.

In summary, for the first time we reported the relatively high viral loads of HCV among voluntary blood donors who were infected with subtype 6a strains. We also correlated the measured viral loads with detected HCV genotypes and the donors' gender. We found that donors infected with genotype 1 and 6 had significantly higher viral loads than those with genotype 2 and 3, and male donors had significantly higher viral loads than female donors. According to these findings, we speculate that higher viral loads of subtype 6a may have conferred its stronger ability for faster dissemination since this subtype has now become increasingly prevalent in China. Our results may provide new insight into HCV transmission, especially for the emerging 6a strains. This information may help design new strategies that can be used for treating patients infected with HCV genotype 6. However, further studies are required in order to confirm the findings from this study.

## Supporting Information

Figure S1 **Phylogenetic trees reconstructed with NS5B region sequences determined among 298 voluntary blood donors** (**A**) **and with E1 region sequences determined by another one voluntary blood donor** (**B**)**, corresponding to the nucleotide numbering of 8276–8615 in the H77 genome.** Percentages in italics represent bootstrap values in 1000 replicates. Scale bar on the bottom shows 0.1 nucleotide substitutions per site. Reference sequences of 1a, 1b, 2a, 3a, and 3b were shown in Genbank accession numbers and each was indicated with a red pie.
(TIF)

Table S1 **Reference sequences of 1a, 1b, 2a, 3a, 3b and 6a was used to reconstruct phylogenetic tree from genebank.**
(DOCX)

Table S2 **299 plasma samples were measured by the CAP/CTM test to detect viral loads of HCV.** The generated data were analyzed using the Amplilink software. The original data was listed as units per milliliter (IU/ml) and was expressed as log_10_ international units per milliliter (log_10_ IU/ml).
(DOC)
