# Supplementary figures and images for: Correlation of Viral Loads with HCV Genotypes: Higher Levels of Virus Were Revealed among Blood Donors Infected with 6a Strains
Source: PLoS One. 2012 Dec 17;7(12):e52467. doi: 10.1371/journal.pone.0052467 (PMC3524124; doi:10.1371/journal.pone.0052467)

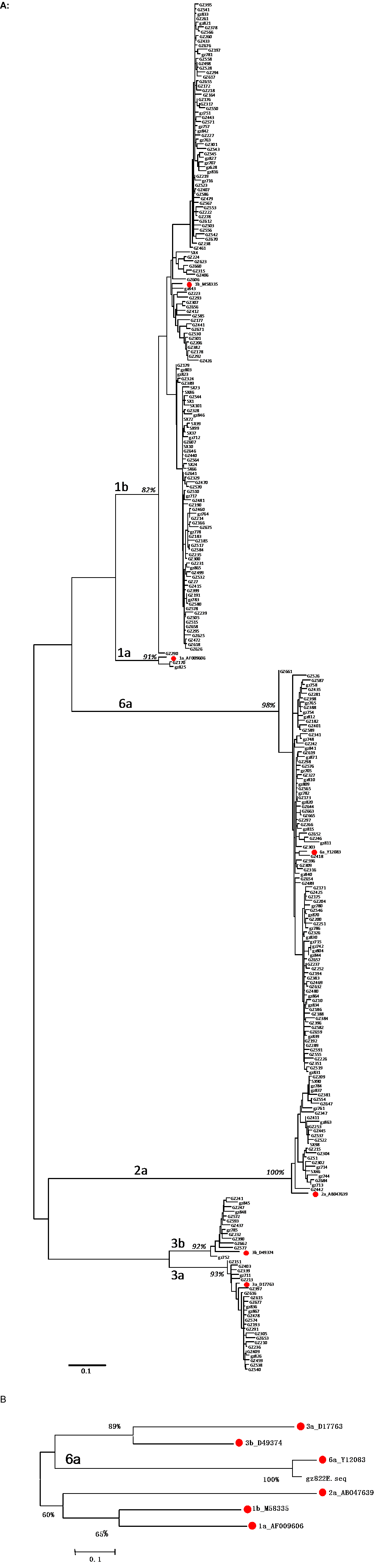

Supplement: Figure S1 — Phylogenetic trees reconstructed with NS5B region sequences determined among 298 voluntary blood donors (A) and with E1 region sequences determined by another one voluntary blood donor (B), corresponding to the nucleotide numbering of 8276–8615 in the H77 genome. Percentages in italics represent bootstrap values in 1000 replicates. Scale bar on the bottom shows 0.1 nucleotide substitutions per site. Reference sequences of 1a, 1b, 2a, 3a, and 3b were shown in Genbank accession numbers and each was indicated with a red pie. (TIF) [file pone.0052467.s001.tif]
