# Supplementary material for: Correlation of Viral Loads with HCV Genotypes: Higher Levels of Virus Were Revealed among Blood Donors Infected with 6a Strains
Source: PLoS One. 2012 Dec 17;7(12):e52467. doi: 10.1371/journal.pone.0052467 (PMC3524124; doi:10.1371/journal.pone.0052467)
Supplement: Table S1 — Reference sequences of 1a, 1b, 2a, 3a, 3b and 6a was used to reconstruct phylogenetic tree from genebank. (DOCX) [file pone.0052467.s002.docx]

Supplementary table S1: Reference sequences of 1a, 1b, 2a, 3a, 3b and 6a.

| Genotype | Genbank accession number |
| --- | --- |
| 1 a | AF009606 |
| 1b | M58335 |
| 2a | AB047639 |
| 3a | D17763 |
| 3b | D49374 |
| 6a | Y12083 |
