# Supplementary material for: Correlation of Viral Loads with HCV Genotypes: Higher Levels of Virus Were Revealed among Blood Donors Infected with 6a Strains
Source: PLoS One. 2012 Dec 17;7(12):e52467. doi: 10.1371/journal.pone.0052467 (PMC3524124; doi:10.1371/journal.pone.0052467)
Supplement: Table S2 — 299 plasma samples were measured by the CAP/CTM test to detect viral loads of HCV. The generated data were analyzed using the Amplilink software. The original data was listed as units per milliliter (IU/ml) and was expressed as log10 international units per milliliter (log10 IU/ml). (DOC) [file pone.0052467.s003.doc]

Supplementary table S2：The raw data of HCV viral loads.

| Number | HCV viral loads(IU/ml) | log_10_ HCV viral loads |
| --- | --- | --- |
| 1 | 1.25E+03 | 3.096910013 |
| 2 | 1.84E+03 | 3.264817823 |
| 3 | 2.04E+03 | 3.309630167 |
| 4 | 2.18E+03 | 3.338456494 |
| 5 | 2.49E+03 | 3.396199347 |
| 6 | 4.36E+03 | 3.639486489 |
| 7 | 5.13E+03 | 3.710117365 |
| 8 | 5.45E+03 | 3.736396502 |
| 9 | 7.88E+03 | 3.896526217 |
| 10 | 8.23E+03 | 3.915399835 |
| 11 | 8.29E+03 | 3.918554531 |
| 12 | 8.51E+03 | 3.92992956 |
| 13 | 9.17E+03 | 3.962369336 |
| 14 | 1.13E+04 | 4.053078443 |
| 15 | 1.14E+04 | 4.056904851 |
| 16 | 1.21E+04 | 4.08278537 |
| 17 | 1.36E+04 | 4.133538908 |
| 18 | 1.39E+04 | 4.1430148 |
| 19 | 1.50E+04 | 4.176091259 |
| 20 | 1.51E+04 | 4.178976947 |
| 21 | 1.52E+04 | 4.181843588 |
| 22 | 1.52E+04 | 4.181843588 |
| 23 | 2.00E+04 | 4.301029996 |
| 24 | 2.18E+04 | 4.338456494 |
| 25 | 2.29E+04 | 4.359835482 |
| 26 | 2.30E+04 | 4.361727836 |
| 27 | 2.51E+04 | 4.399673721 |
| 28 | 2.58E+04 | 4.411619706 |
| 29 | 2.59E+04 | 4.413299764 |
| 30 | 2.76E+04 | 4.440909082 |
| 31 | 2.92E+04 | 4.465382851 |
| 32 | 3.07E+04 | 4.487138375 |
| 33 | 3.08E+04 | 4.488550717 |
| 34 | 3.21E+04 | 4.506505032 |
| 35 | 3.28E+04 | 4.515873844 |
| 36 | 3.35E+04 | 4.525044807 |
| 37 | 3.45E+04 | 4.537819095 |
| 38 | 3.83E+04 | 4.583198774 |
| 39 | 4.27E+04 | 4.630427875 |
| 40 | 4.35E+04 | 4.638489257 |
| 41 | 5.17E+04 | 4.713490543 |
| 42 | 5.65E+04 | 4.752048448 |
| 43 | 6.60E+04 | 4.819543936 |
| 44 | 6.95E+04 | 4.841984805 |
| 45 | 7.16E+04 | 4.854913022 |
| 46 | 7.22E+04 | 4.858537198 |
| 47 | 8.07E+04 | 4.906873535 |
| 48 | 9.08E+04 | 4.958085849 |
| 49 | 9.16E+04 | 4.961895474 |
| 50 | 9.47E+04 | 4.976349979 |
| 51 | 1.09E+05 | 5.037426498 |
| 52 | 1.13E+05 | 5.053078443 |
| 53 | 1.33E+05 | 5.123851641 |
| 54 | 1.34E+05 | 5.127104798 |
| 55 | 1.34E+05 | 5.127104798 |
| 56 | 1.39E+05 | 5.1430148 |
| 57 | 1.56E+05 | 5.193124598 |
| 58 | 1.61E+05 | 5.206825876 |
| 59 | 1.66E+05 | 5.220108088 |
| 60 | 1.68E+05 | 5.225309282 |
| 61 | 1.72E+05 | 5.235528447 |
| 62 | 1.74E+05 | 5.240549248 |
| 63 | 1.81E+05 | 5.257678575 |
| 64 | 1.85E+05 | 5.267171728 |
| 65 | 1.88E+05 | 5.274157849 |
| 66 | 2.37E+05 | 5.374748346 |
| 67 | 2.45E+05 | 5.389166084 |
| 68 | 2.49E+05 | 5.396199347 |
| 69 | 2.51E+05 | 5.399673721 |
| 70 | 2.54E+05 | 5.404833717 |
| 71 | 2.65E+05 | 5.423245874 |
| 72 | 3.24E+05 | 5.51054501 |
| 73 | 3.25E+05 | 5.511883361 |
| 74 | 3.61E+05 | 5.557507202 |
| 75 | 3.74E+05 | 5.572871602 |
| 76 | 3.88E+05 | 5.588831726 |
| 77 | 4.15E+05 | 5.618048097 |
| 78 | 4.39E+05 | 5.64246452 |
| 79 | 4.43E+05 | 5.646403726 |
| 80 | 4.52E+05 | 5.655138435 |
| 81 | 4.56E+05 | 5.658964843 |
| 82 | 4.70E+05 | 5.672097858 |
| 83 | 4.70E+05 | 5.672097858 |
| 84 | 4.72E+05 | 5.673941999 |
| 85 | 4.72E+05 | 5.673941999 |
| 86 | 4.89E+05 | 5.689308859 |
| 87 | 5.12E+05 | 5.709269961 |
| 88 | 5.15E+05 | 5.711807229 |
| 89 | 5.50E+05 | 5.740362689 |
| 90 | 5.52E+05 | 5.741939078 |
| 91 | 5.59E+05 | 5.747411808 |
| 92 | 5.92E+05 | 5.772321707 |
| 93 | 6.01E+05 | 5.778874472 |
| 94 | 6.26E+05 | 5.796574333 |
| 95 | 6.29E+05 | 5.798650645 |
| 96 | 6.29E+05 | 5.798650645 |
| 97 | 6.54E+05 | 5.815577748 |
| 98 | 6.84E+05 | 5.835056102 |
| 99 | 7.10E+05 | 5.851258349 |
| 100 | 7.34E+05 | 5.86569606 |
| 101 | 7.37E+05 | 5.867467488 |
| 102 | 7.46E+05 | 5.872738827 |
| 103 | 7.50E+05 | 5.875061263 |
| 104 | 7.89E+05 | 5.897077003 |
| 105 | 7.96E+05 | 5.900913068 |
| 106 | 8.04E+05 | 5.905256049 |
| 107 | 8.14E+05 | 5.910624405 |
| 108 | 8.18E+05 | 5.912753304 |
| 109 | 8.27E+05 | 5.91750551 |
| 110 | 8.50E+05 | 5.929418926 |
| 111 | 8.56E+05 | 5.932473765 |
| 112 | 8.71E+05 | 5.940018155 |
| 113 | 9.11E+05 | 5.959518377 |
| 114 | 9.15E+05 | 5.961421094 |
| 115 | 9.17E+05 | 5.962369336 |
| 116 | 9.23E+05 | 5.965201701 |
| 117 | 9.30E+05 | 5.968482949 |
| 118 | 9.53E+05 | 5.979092901 |
| 119 | 9.63E+05 | 5.983626287 |
| 120 | 1.01E+06 | 6.004321374 |
| 121 | 1.02E+06 | 6.008600172 |
| 122 | 1.11E+06 | 6.045322979 |
| 123 | 1.13E+06 | 6.053078443 |
| 124 | 1.13E+06 | 6.053078443 |
| 125 | 1.17E+06 | 6.068185862 |
| 126 | 1.22E+06 | 6.086359831 |
| 127 | 1.24E+06 | 6.093421685 |
| 128 | 1.24E+06 | 6.093421685 |
| 129 | 1.26E+06 | 6.100370545 |
| 130 | 1.27E+06 | 6.103803721 |
| 131 | 1.30E+06 | 6.113943352 |
| 132 | 1.43E+06 | 6.155336037 |
| 133 | 1.44E+06 | 6.158362492 |
| 134 | 1.46E+06 | 6.164352856 |
| 135 | 1.48E+06 | 6.170261715 |
| 136 | 1.49E+06 | 6.173186268 |
| 137 | 1.52E+06 | 6.181843588 |
| 138 | 1.56E+06 | 6.193124598 |
| 139 | 1.61E+06 | 6.206825876 |
| 140 | 1.63E+06 | 6.212187604 |
| 141 | 1.64E+06 | 6.214843848 |
| 142 | 1.64E+06 | 6.214843848 |
| 143 | 1.71E+06 | 6.23299611 |
| 144 | 1.72E+06 | 6.235528447 |
| 145 | 1.79E+06 | 6.252853031 |
| 146 | 1.80E+06 | 6.255272505 |
| 147 | 1.88E+06 | 6.274157849 |
| 148 | 1.92E+06 | 6.283301229 |
| 149 | 1.93E+06 | 6.285557309 |
| 150 | 1.96E+06 | 6.292256071 |
| 151 | 1.98E+06 | 6.29666519 |
| 152 | 2.01E+06 | 6.303196057 |
| 153 | 2.05E+06 | 6.311753861 |
| 154 | 2.06E+06 | 6.31386722 |
| 155 | 2.08E+06 | 6.318063335 |
| 156 | 2.13E+06 | 6.328379603 |
| 157 | 2.13E+06 | 6.328379603 |
| 158 | 2.18E+06 | 6.338456494 |
| 159 | 2.21E+06 | 6.344392274 |
| 160 | 2.22E+06 | 6.346352974 |
| 161 | 2.24E+06 | 6.350248018 |
| 162 | 2.26E+06 | 6.354108439 |
| 163 | 2.30E+06 | 6.361727836 |
| 164 | 2.36E+06 | 6.372912003 |
| 165 | 2.38E+06 | 6.376576957 |
| 166 | 2.43E+06 | 6.385606274 |
| 167 | 2.44E+06 | 6.387389826 |
| 168 | 2.47E+06 | 6.392696953 |
| 169 | 2.50E+06 | 6.397940009 |
| 170 | 2.52E+06 | 6.401400541 |
| 171 | 2.53E+06 | 6.403120521 |
| 172 | 2.58E+06 | 6.411619706 |
| 173 | 2.58E+06 | 6.411619706 |
| 174 | 2.61E+06 | 6.416640507 |
| 175 | 2.68E+06 | 6.428134794 |
| 176 | 2.69E+06 | 6.42975228 |
| 177 | 2.69E+06 | 6.42975228 |
| 178 | 2.75E+06 | 6.439332694 |
| 179 | 2.80E+06 | 6.447158031 |
| 180 | 2.81E+06 | 6.44870632 |
| 181 | 2.85E+06 | 6.45484486 |
| 182 | 2.87E+06 | 6.457881897 |
| 183 | 2.88E+06 | 6.459392488 |
| 184 | 2.90E+06 | 6.462397998 |
| 185 | 2.94E+06 | 6.46834733 |
| 186 | 2.96E+06 | 6.471291711 |
| 187 | 3.01E+06 | 6.478566496 |
| 188 | 3.03E+06 | 6.481442629 |
| 189 | 3.06E+06 | 6.485721426 |
| 190 | 3.09E+06 | 6.489958479 |
| 191 | 3.13E+06 | 6.495544338 |
| 192 | 3.14E+06 | 6.496929648 |
| 193 | 3.15E+06 | 6.498310554 |
| 194 | 3.18E+06 | 6.50242712 |
| 195 | 3.22E+06 | 6.507855872 |
| 196 | 3.24E+06 | 6.51054501 |
| 197 | 3.26E+06 | 6.5132176 |
| 198 | 3.30E+06 | 6.51851394 |
| 199 | 3.31E+06 | 6.519827994 |
| 200 | 3.33E+06 | 6.522444234 |
| 201 | 3.34E+06 | 6.523746467 |
| 202 | 3.41E+06 | 6.532754379 |
| 203 | 3.52E+06 | 6.546542663 |
| 204 | 3.54E+06 | 6.549003262 |
| 205 | 3.54E+06 | 6.549003262 |
| 206 | 3.56E+06 | 6.551449998 |
| 207 | 3.66E+06 | 6.563481085 |
| 208 | 3.70E+06 | 6.568201724 |
| 209 | 3.80E+06 | 6.579783597 |
| 210 | 3.91E+06 | 6.592176757 |
| 211 | 4.08E+06 | 6.610660163 |
| 212 | 4.11E+06 | 6.613841822 |
| 213 | 4.25E+06 | 6.62838893 |
| 214 | 4.29E+06 | 6.632457292 |
| 215 | 4.30E+06 | 6.633468456 |
| 216 | 4.31E+06 | 6.63447727 |
| 217 | 4.45E+06 | 6.648360011 |
| 218 | 4.47E+06 | 6.650307523 |
| 219 | 4.51E+06 | 6.654176542 |
| 220 | 4.58E+06 | 6.660865478 |
| 221 | 4.66E+06 | 6.668385917 |
| 222 | 4.71E+06 | 6.673020907 |
| 223 | 4.71E+06 | 6.673020907 |
| 224 | 4.72E+06 | 6.673941999 |
| 225 | 4.72E+06 | 6.673941999 |
| 226 | 4.82E+06 | 6.683047038 |
| 227 | 4.86E+06 | 6.686636269 |
| 228 | 5.03E+06 | 6.701567985 |
| 229 | 5.20E+06 | 6.716003344 |
| 230 | 5.28E+06 | 6.722633923 |
| 231 | 5.34E+06 | 6.727541257 |
| 232 | 5.38E+06 | 6.730782276 |
| 233 | 5.50E+06 | 6.740362689 |
| 234 | 5.54E+06 | 6.743509765 |
| 235 | 5.61E+06 | 6.748962861 |
| 236 | 5.65E+06 | 6.752048448 |
| 237 | 5.66E+06 | 6.752816431 |
| 238 | 5.71E+06 | 6.756636108 |
| 239 | 5.75E+06 | 6.759667845 |
| 240 | 6.07E+06 | 6.783188691 |
| 241 | 6.26E+06 | 6.796574333 |
| 242 | 6.30E+06 | 6.799340549 |
| 243 | 6.33E+06 | 6.80140371 |
| 244 | 6.46E+06 | 6.810232518 |
| 245 | 6.52E+06 | 6.814247596 |
| 246 | 6.52E+06 | 6.814247596 |
| 247 | 6.55E+06 | 6.8162413 |
| 248 | 6.67E+06 | 6.824125834 |
| 249 | 6.73E+06 | 6.828015064 |
| 250 | 6.78E+06 | 6.831229694 |
| 251 | 6.88E+06 | 6.837588438 |
| 252 | 6.88E+06 | 6.837588438 |
| 253 | 6.92E+06 | 6.840106094 |
| 254 | 6.98E+06 | 6.843855423 |
| 255 | 7.08E+06 | 6.850033258 |
| 256 | 7.11E+06 | 6.851869601 |
| 257 | 7.17E+06 | 6.855519156 |
| 258 | 7.17E+06 | 6.855519156 |
| 259 | 7.37E+06 | 6.867467488 |
| 260 | 7.37E+06 | 6.867467488 |
| 261 | 7.43E+06 | 6.870988814 |
| 262 | 7.44E+06 | 6.871572936 |
| 263 | 7.49E+06 | 6.874481818 |
| 264 | 7.50E+06 | 6.875061263 |
| 265 | 7.59E+06 | 6.880241776 |
| 266 | 7.69E+06 | 6.88592634 |
| 267 | 7.74E+06 | 6.888740961 |
| 268 | 7.93E+06 | 6.899273187 |
| 269 | 7.96E+06 | 6.900913068 |
| 270 | 8.03E+06 | 6.904715545 |
| 271 | 8.05E+06 | 6.90579588 |
| 272 | 8.10E+06 | 6.908485019 |
| 273 | 8.15E+06 | 6.911157609 |
| 274 | 8.95E+06 | 6.951823035 |
| 275 | 8.99E+06 | 6.953759692 |
| 276 | 9.18E+06 | 6.962842681 |
| 277 | 9.52E+06 | 6.978636948 |
| 278 | 1.09E+07 | 7.037426498 |
| 279 | 1.14E+07 | 7.056904851 |
| 280 | 1.24E+07 | 7.093421685 |
| 281 | 1.25E+07 | 7.096910013 |
| 282 | 1.27E+07 | 7.103803721 |
| 283 | 1.31E+07 | 7.117271296 |
| 284 | 1.35E+07 | 7.130333768 |
| 285 | 1.35E+07 | 7.130333768 |
| 286 | 1.39E+07 | 7.1430148 |
| 287 | 1.71E+07 | 7.23299611 |
| 288 | 1.82E+07 | 7.260071388 |
| 289 | 1.90E+07 | 7.278753601 |
| 290 | 1.93E+07 | 7.285557309 |
| 291 | 1.96E+07 | 7.292256071 |
| 292 | 2.02E+07 | 7.305351369 |
| 293 | 2.03E+07 | 7.307496038 |
| 294 | 2.58E+07 | 7.411619706 |
| 295 | 2.66E+07 | 7.424881637 |
| 296 | 3.07E+07 | 7.487138375 |
| 297 | 3.57E+07 | 7.552668216 |
| 298 | 4.72E+07 | 7.673941999 |
| 299 | 6.76E+07 | 7.829946696 |
